# Supplementary material for: On the origin of nonequivalent states: How we can talk about preprints
Source: F1000Res. 2017 May 2;6:608. [Version 1] doi: 10.12688/f1000research.11408.1 (PMC5461893; doi:10.12688/f1000research.11408.1)
Supplement: Peer review history

# Peer review: Referee response for version 1

**DOI:** 10.5256/f1000research.12315.r22413

**Published:** 2017-05-30

**Authors:** Todd Carpenter

**Affiliations:** 1 National Information Standards Organization (NISO), Baltimore, MD, USA

There are many challenges in our current scholarly communications and assessment environment.  This article draws out an important distinction between the objective status of a piece of scholarly content and the value to which the community assigns to that content. This is an intricate, intertwined, and sometimes confusing interplay between the two concepts. The authors do a commendable job of describing the current state and outline potentially valuable model for distinguishing between the two concepts.

 While the article does have much to recommend, I have several areas of concern. First, the article provides a quaint description of two scholars in two distinctly different fields of inquiry, physics and biology, who collaborate on a research project. One of the researchers, the physicist, receives "credit" for a joint paper, while the other, the naturalist, receives none. The article takes the reader on a journey where one researcher succeeds while the other fails because of a lack distinction between the two social responses to the same content form. This illustration describes the sometimes critical differences between domains and the different weight given to forms of distribution and publication.  In the article's conclusion, the authors note that their framework isn't meant to address the social differences between domains that are at the root of these differences, simply to describe them. While distinguishing between “state” and “standing” might provide some method to identify the objective and subjective status of a content object, the article lacks consideration of the criteria or suggestions about what characteristics might contribute to their notion of standing. At the heart of the illustration is an environment where different domains confer different meaning or value, the objective status may or may not influence the subjective response. Distinguishing between the two seems obvious.

 While the distinction between "State" and "Standing" as described in the framework appears a useful distinguishing characteristic, it is not clear to me that the examples of "state" changes are in fact "objectively determinable characteristics of the object" that are intrinsic to the object itself.  There is no way to know by examining the object whether it has undergone any particular state change. To consider a real world  example, take an article in ArXiv ( https://arxiv.org/abs/1509.06859v2) by Sébastien Gouëzel (LMJL).  This paper has an earlier version ( https://arxiv.org/abs/1509.06859v1) and was updated with the current version in May, 2017.  Viewing this from ArXiv, there is no indication that this object has gone through any vetting, nor any editorial review, nor any validation processes, nor any copy editing, nor any of the other state changes mentioned in this paper.  However, the paper has been included in the online journal *Discrete Analysis* .  There is an editorial introduction with a DOI (10.19086/da.1639, which oddly didn't resolve) and it isn't clear that the journal "publishes" the article or the introductions. Presumably the article itself went through a peer review, an editorial review, and possibly edited and then was revised and resubmitted to ArXiv. The date on the ArXiv revision is 5 May 2017, four days before the Discrete Analysis paper was posted on 9 May 2017.  If a user views this article through the wrapper of journal, it may be clear that these "state changes" as defined in this paper might apply, but the same content viewed through the lens of the paper directly on ArXiv they are not.  The state changes exist in one environment but not in another.

 The authors respond to this situation, as they note in their conclusion, that this is simply a failing of metadata and that if only the state changes were recorded in the metadata, this problem might be addressed. The problems of metadata quality is well known and much discussed in the community. Properly assigning metadata to a final version of record is challenging enough, without retroactively populating metadata or ensuring a string of provenance data is included with the current object to support this chain of awareness of the current state of a content object.  For example, if someone were to come across the first version of Gouëzel's paper in the example I noted, how would anyone know the current state of the previous version?  Without forward linking, there is no way to know that the preprint version (or authors original, using the JAV terminology) was followed by another version?

 With this example in mind, the paper would be strengthened through a more robust description of state changes, and what would distinguish a state change from something less substantial. Since many of the changes that would might take place to an article may or may not be significant. Also, many state changes might not lead to notable changes. For example, (in a closed peer-review environment say), I may have read this article without recommending any changes. The act of reading and saying "Yes, this is OK", is completely external to the object and failing quality metadata to describe the review. These external acts related to a piece of content are critical to the process of developing standing, but aren't necessarily externally obvious, as the authors note.

 A minor point about standing to which I quibble is the notion that standing is not something that can be conferred individually. There are many instances when standing could be individually confirmed.  Many journals are editorially run by a single individual, who might review, take a decision to publish or not, or approve for publication. A department chair, may determine that a piece of content is appropriate for inclusion in a promotion or tenure decision.  I am sure there are countless other examples of this.  One might say the editor is speaking on behalf of a community of subscribers, but in reality it is just one person taking the decision.

 In practice, the framework outline in this article builds upon the structure outlined in the NISO Journal Article Versions (NISO JAV) Recommended Practice, which defined a structure of changes for the constrained scope of journal articles. That effort settled by "identif[ing] a significant value-added “state change” in the progress of a journal article from origination to publication." While these state changes in NISO JAV are explicitly focused on the formal publication process, the concept of state change applies across all forms of content, again as noted in the introduction of NISO JAV. It should also be noted that the working group that developed NISO JAV structure intentionally did not extend it's scope to other forms of content, nor to extend the resulting vocabulary to every instance in the content creation process.

 This article, or potentially in subsequent work by the authors, would be strengthened by a discussion of the types of elements that go into standing. The description of potential changes to a content object's "state" are comparatively robust, but the components of what constitutes "standing" is decidedly weaker.  Especially since this appears to be the core argument of the need for this framework, this lack of discussion around those details glosses over the difficulty in that side of this environment.

 Inherently, this second domain of the meaning of and definitions of "standing" are incredibly fraught and fungible across the academy. What has standing in one domain does not in another, often without rhyme or reason. There is no fault in the authors avoiding these very granular and thorny questions in this article, nor does it diminish from the value in trying to distinguish between the two.  However, without the understanding of "standing" there can be no resolution to the problems that Darwin faces in the article's opening illustration.

 I look forward to the continued discussion around these issues and encourage the authors to continue to develop their work in this area.

I have read this submission. I believe that I have an appropriate level of expertise to confirm that it is of an acceptable scientific standard.

# Peer review: Referee response for version 1

**DOI:** 10.5256/f1000research.12315.r22411

**Published:** 2017-05-19

**Authors:** Kathleen Fitzpatrick

**Affiliations:** 1 Modern Language Association, New York, NY, USA

This article focuses on the need (most immediately seen in recent discussions of the challenges presented by "preprints") for distinguishing between the ways we represent the state of research outputs and the ways we represent the standing those outputs have in accordance with that state. The importance of this distinction, as the authors point out, is in recognizing that different communities of practice accord different value to outputs that are objectively in the same state. By “separating the description of process (and testing whether the stated process has been followed) from the views of any given community of objects that have been through that process” (5), we might be better able to speak across disciplinary boundaries about the value of the work we do. The distinction between state and standing is especially crucial for those who seek to change scholarly communication practices, for instance by allowing a greater range of outputs to “count” in hiring and assessment processes, in order to make clear that the transformation that matters lies in “the social machinery of state-standing links” (5). By encouraging the disambiguation of state and standing, the authors are able to advise publishers, platforms, and scholarly communities on ways they might contribute to better conversations about the value of particular research outputs.

 The article begins with a highly engaging opening illustration of the stakes of the non-universality not only of language but of practices in scholarly communication, and continues through careful and well-documented argumentation. The authors look carefully at distinctions not just in terminology but also in values across different fields. They are careful to note that they are not recommending a new vocabulary, nor a guiding framework for how scholarly communities should negotiate the current changes in their communication practices, but they do our fields a great service by exposing the reasons for much of our mutual incomprehension across fields. They also go a long way toward explaining why “we have to make preprints ‘count’” is a very heavy lift in some communities.

 I do hope that the authors will continue their research in this line. It would be great to have their input, for instance, into the construction of metadata that can help clarify changes in a research object’s state, enabling better judgment in communities about its standing.

 A few very small copyediting notes:

- The phrase “different to” is used a couple of times; I’m honestly not sure if this is a UK/US distinction, but I’d argue for “different from” instead.
- On page 3, column 2, line 1, “that make” should be “that makes”.
- On page 4, AOP is glossed as “advance online publication” in the caption for Figure 1, and “Advanced Online Publication” in the text.

I am grateful for the opportunity to have reviewed this article, and I look forward to the discussions that it might inspire.

I have read this submission. I believe that I have an appropriate level of expertise to confirm that it is of an acceptable scientific standard.
